# Prevalence and associated factors of adolescent pregnancy (15–19 years) in East Africa: a multilevel analysis

**DOI:** 10.1186/s12884-021-03713-9

**Published:** 2021-03-26

**Authors:** Misganaw Gebrie Worku, Zemenu Tadesse Tessema, Achamyeleh Birhanu Teshale, Getayeneh Antehunegn Tesema, Yigizie Yeshaw

**Affiliations:** 1 Department of Human Anatomy, College of Medicine and Health Science, School of Medicine, University of Gondar, Gondar, Ethiopia; 2 Department of Epidemiology and Biostatistics, Institute of Public Health, College of Medicine and Health Sciences, University of Gondar, Gondar, Ethiopia; 3 Department of Human Physiology, College of Medicine and Health Science, School of Medicine, University of Gondar, Gondar, Ethiopia

**Keywords:** Adolescent pregnancy, East Africa, Multilevel analysis

## Abstract

**Background:**

Adolescent pregnancy is a major public health problem both in developed and developing countries with huge consequences to maternal health and pregnancy outcomes. However, there is limited evidence on the prevalence and associated factors of adolescent pregnancy in East Africa. Therefore, this study aimed to investigate the prevalence and associated factors of adolescent pregnancy in Eastern Africa.

**Method:**

The most recent Demographic and Health Survey (DHS) datasets of the 12 East African countries were used. A total weighted sample of 17, 234 adolescent girls who ever had sex was included. A multilevel binary logistic regression analysis was fitted to identify the significantly associated factors of adolescent pregnancy. Finally, the Adjusted Odds Ratio (AOR) with 95% Confidence Interval (CI) were reported to declare the factors that are significantly associated with adolescent pregnancy.

**Results:**

The overall prevalence of adolescent pregnancy in East Africa was 54.6% (95%CI: 53.85, 55.34%). In the multivariable multilevel analysis; being age 18–19 years [AOR = 3.06; 95%CI: 2.83, 3.31], using contraceptive [AOR = 1.41; 95%CI: 1.28, 1.55], being employed girls [AOR = 1.11; 95%CI: 1.03, 1.19], being spouse/head within the family [AOR = 1.62; 95% CI: 1.45, 1.82], and being from higher community level contraceptive utilization [AOR = 1.10; 95%CI:1.02, 1.19] were associated with higher odds of adolescent pregnancy. While adolescent girls attained secondary education and higher [AOR = 0.78; 95%CI: 0.68, 0.91], initiation of sex at age of 15 to 14 years [AOR = 0.69; 95%CI: 0.63, 0.75] and 18 to 19 years [AOR = 0.31; 95%CI: 0.27, 0.35], being unmarried [AOR = 0.25; 95%CI: 0.23, 0.28], having media exposure [AOR = 0.85; 95%CI: 0.78, 0.92], and being girls from rich household [AOR = 0.64; 95%CI: 0.58, 0.71] were associated with lower odds of adolescent pregnancy.

**Conclusion:**

This study found that adolescent pregnancy remains a common health care problem in East Africa. Age, contraceptive utilization, marital status, working status, household wealth status, community-level contraceptive utilization, age at initiation of sex, media exposure, educational level and relation to the household head were associated with adolescent pregnancy. Therefore, designing public health interventions targeting higher risk adolescent girls such as those from the poorest household through enhancing maternal education and empowerment is vital to reduce adolescent pregnancy and its complications.

## Background

Adolescent pregnancy is a global public health problem that affects both developed and developing countries [1]. Nearly 25% of adolescent women have got pregnant worldwide [1–3], and the prevalence of adolescent pregnancy in Africa is 18.8%, of this, 19.3% occurred in Sub-Saharan Africa and 21.5% in eastern Africa [2]. The prevalence of adolescent pregnancy in eastern Africa ranges from 18 to 29% and around half of these pregnancies are unintended [4].

Globally an estimated 3.9 million adolescents experience unsafe abortions, which contribute to the highest maternal mortality and morbidity [5, 6]. Adolescent pregnancy is considered the leading cause of newborn and maternal mortality in developing countries [7–9]. Pregnancies among adolescents are associated with several adverse health, educational, social and economic outcomes [10, 11]. Adolescent pregnancies typically occur in poor populations, which could be influenced by poverty, lack of education, and work opportunities [12].

Adolescent pregnancy has significant health, psychological and socioeconomic impacts on the mother. It increases the risk of low birth weight, premature delivery, mortality, preeclampsia, social isolation, delayed or neglected educational goals, and maternal depression [8]. The social consequence includes stigma, rejection, violence and drops out of school [13, 14]. Due to their direct association with adolescent sexual intercourse, several biological factors such as the timing of pubertal development, hormone levels, and genes, are also related to adolescent pregnancy [15]. In general, adolescent mothers had a low level of education and low level of antenatal care and faces a higher risk of developing pregnancy-induced hypertension (PIH), Preeclampsia toxemia [16], eclampsia, premature labor onset, and premature delivery with increased risk of neonatal morbidity and mortality [3, 17, 18].

Previous studies showed that being sexually active at an early age, early marriage, older teenage, married women, educational attainment, age at 1st sex, household wealth, family structure, exposure to media, community poverty level, and contraceptive use are significantly associated with adolescent pregnancy [3, 4, 13, 19, 20].

Though there are studies conducted on the prevalence and associated factors of adolescent pregnancy in individual east African countries [4, 7–9, 16], there is limited evidence on the pooled prevalence and associated factors of adolescent pregnancy in the region. Therefore, this study aimed to determine the pooled prevalence and associated factors of adolescent pregnancy in East Africa based on the pooled nationally representative Demographic and Health Surveys (DHS). Thus, the findings of this study could help policymakers, and governmental and non-governmental organizations to design programs and interventions towards adolescent pregnancy and pregnancy-related complications.

## Methods

### Data sources, sampling technique, and study population

This study was a secondary data analysis based on the datasets from the most recent Demographic and Health Surveys (DHS) conducted in East African countries (Burundi, Ethiopia, Comoros, Uganda, Rwanda, Tanzania, Mozambique, Madagascar, Zimbabwe, Kenya, Zambia, and Malawi). These datasets were appended to determine the prevalence and associated factors of adolescent pregnancy in east Africa. The DHS is a nationally representative survey that collects data on basic health indicators like mortality, morbidity, family planning service utilization, fertility, maternal and child health. The DHS used two stage stratified sampling technique to select the study participants. Each country’s survey consists of different datasets including men, women, children, birth, and household datasets, and for this study, we used the women’s dataset (individual record (IR) file). In this study, all adolescent girls aged 15–19 years and those who ever had sex (a total weighted sample of 17, 234) were considered for the final analysis. The detailed information on the survey country, the number of adolescents in each country, eligible and actual number of women for each country were provided in Table 1.

**Table 1:** Survey and sample size characteristics

Country	Year of survey	Total adolescent girls interviewed	Sample size by design	Selected adolescent girls	% of completed responses
Burundi	2016	3968	562	562	100%
Ethiopia	2016	3498	905	905	100%
Kenya	2014	6078	2233	2228	99.77%
Comoros	2012	1295	285	285	100%
Madagascar	2008	4034	2223	2223	100%
Malawi	2015/16	5273	2670	2670	100%
Mozambique	2011	3065	2044	2044	100%
Rwanda	2352	2779	558	558	100%
Tanzania	2015/16	2932	1356	1356	100%
Uganda	2016	4276	1957	1956	99.94%
Zambia	2018	3686	1883	1879	99.78%
Zimbabwe	2013/2014	2156	708	708	100%
Total		43,040^a^	17,384^a^	17,374^a^	99.94%

Note: ^a^ = Unweighted frequency

### Variables of the study

The outcome variable of this study was “getting pregnant during the age of 15-19 years among adolescents who ever had sex”. A woman was considered as experiencing adolescent/teenage pregnancy if her age was from 15 to 19 and if she had ever been pregnant before or during the survey. We used all girls age 15–19 who had ever experienced sex as our study population. The outcome was derived using the variables; the number of women who have had a birth and the number of women who have not had a birth but are pregnant at the time of interview [14].

The independent variables considered for this study were both individual and community-level variables. The individual-level factors include; the age of respondent, marital status, age at 1st sex, contraceptive use, educational attainment, household wealth status, sex of household head, relation to household head, and access to mass media. The community-level factors were community women education, community poverty, community contraceptive utilization, residence and country. In DHS, except country and residence, all the other variables were collected at the individual level. Therefore, we generate three community-level variables such as community women’s education, community poverty, and community contraceptive utilization by aggregating the individual-level factors at cluster level and categorized as high and low based on the median value (Table 2).

**Table 2:** Description and measurement of independent variables

Independent variables and their description/categorization	
Individual level variables	
Age Group	Current age of the women and re-coded in to two categories with values of “0” for 15–17, “1” for 18–19.
Wealth Index	The datasets contained wealth index that was created using principal components analysis coded as “poorest”, “poorer”, “Middle”, “Richer”, and “Richest in the EDHS data set.” For this study we recoded it in to three categories as “poor” (includes the poorest and the poorer categories), “middle”, and “rich” (includes the richer and the richest categories)
Occupation	Re-coded in two categories with a value of “0” for not working, and “1” for working.
Media exposure	A composite variable obtained by combining whether a respondent reads newspaper/magazine, listen to radio, and watch television with a value of “0” if a women were not exposed to at least one of the three medias, and “1” if a woman has access/exposure to at least one of the three medias.
Educational status	This is the minimum educational level a woman achieved and re-coded in to three groups with a value of “0” for no education, “1” for primary education, and “2” for secondary and above (combining secondary and higher education categories together).
Marital status	This was the current marital status of women and recoded in two categories with a value of “0” for unmarried (includes those who were never in union, divorced, widowed, and separated), and “1” for “married” (includes those living with partner and those who are married)
Sex of household	The variable sex of household head was recorded as male and female in the dataset and we used without change.
Relation to house hold head	The variable relation to house hold head was recoded as “0” for head/spouse, “1” for Daughter and “2” for relative or other based on DHS guide.
Age at first sex	The variable age at first sex was recoded as “0” for women who initiate sex at age of 5–14 “1” for initiation of sex at 15–17 “2” for women who initiate sex at the age of 18–19.
Contraceptive usage	Recoded in to two categories with value of 0 for “no” if a women don’t use any of the contraceptive methods, and 1 for “Yes” if a women use any of the contraceptive methods. of either of or combination of the following methods(female sterilization, male sterilization, contraceptive pill, intrauterine contraceptive device, injectable, implants, female condom, male condom, diaphragm, contraceptive foam and contraceptive jelly, lactational amenorrhea method, standard days method, and respondent-mentioned other modern contraceptive methods (including cervical cap, contraceptive sponge,)
Community level variables	
Community of poverty level	Measured by proportion of households in the poor (combination of poorer and poorest) wealth quintile derived from data on wealth index. Then it was categorized based on national median value as: low (communities in which < 50% of women had poor socioeconomic status) and high (communities in which ≥50% of women had poor socioeconomic status) poverty level.
Community educational level	Measured by the proportion of educated women (combination of primary, secondary and higher education). It was categorized based on national media value as: low (community in which > 50% of women had no education) and high (community with > 50% of women had utilization of any of the contraceptive methods).
Community contraceptive utilization	Measured by using the proportion of women who used any or the combination of the contraceptives. It was categorized based on national media value as: low (community in which > 50% of women didn’t use any contraceptive methods) and high (community with > 50% of women had education attainment).
Type of place of residence	The variable place of residence recorded as rural and urban in the dataset was used without change.

### Data management and analysis

Data extraction, recoding and analysis were done using STATA version 14 software. The data were weighted before any statistical analysis to restore the representativeness of the data and to get a reliable estimate and standard error. Descriptive statistics were done using frequencies and percentages. Since the DHS data has a hierarchical structure, this violates the independent assumptions of the standard logistic regression model, a multilevel logistic regression analysis was used. Besides, adolescents in the same cluster are more likely to be similar to each other than adolescents from another cluster. This implies that there is a need to take in to account the between cluster variability by using advanced models such as multilevel analysis. The Interclass Correlation Coefficient (ICC) and Median Odds Ratio (MOR) were checked to assess whether there was clustering or not. In this study, four models were fitted; the null model- a model without explanatory variables, model I- a model with individual-level factors, model II- a model with community-level factors, and model III- a model with both individual and community-level factors, simultaneously. Model comparison was done based on deviance (−2LL) and a model with the lowest deviance was selected as the best-fitted model. Both bivariable and multivariable analysis was done using the best-fitted model. At the bivariable analysis variables with a *p*-value ≤0.2 were considered for multivariable analysis. Finally, variables with a *P*-value of ≤0.05 in the multivariable analysis were considered a significant factor associated with adolescent pregnancy.

## Results

### Socio-demographic characteristics

A total of 17, 234 (weighted) adolescent girls who ever had sex was included for the final analysis. Nearly three-fourths (73.78%) of the respondent were rural dwellers and more than half (59.54%) of the respondents were aged 18 to 19 years. About 57.64% of respondents had attained primary education and 39.48% of respondents were from rich households. The majority (70.43%) of adolescent girls had media exposure and 69.51% of respondents were from male-headed households. More than half (53.54%) of adolescent girls were unmarried and 76.92% of adolescent girls did not use any contraceptive (Table 3).

**Table 3:** Sociodemographic characteristics of study participants

Variables		Weighted frequency (%)
Age of respondent	15–17	6977 (40.46%)
	18–19	10,268 (59.54%)
Wealth status	Poor	6998 (40.58%)
	Middle	3440 (19.95%)
	Rich	6808 (39.48%)
Sex of house hold head	Male	11,986 (69.51%)
	Female	5259 (30.49%)
Residence	Rural	12,724 (73.78%)
	Urban	4521 (26.22%)
Media exposure	Yes	12,145 (70.43%)
	No	5101 (29.57%)
Marital statues	Married	8012 (46.46%)
	Unmarried	9233 (53.54%)
Contraceptive use	Yes	3980 (23.08%)
	No	13,265 (76.92%)
Relation to house hold head	Head/spouse	5625 (32.62%)
	Daughter	6106 (35.41%)
	Relative or other	5512 (31.96%)
Respondent working statues	Working	8603 (49.89%)
	Not working	8642 (50.11%)
Educational level	No education	1428 (8.28%)
	Primary education	9938 (57.64%)
	Secondary education	5876 (34.08%)
Age at first sex	5–14	4555 (26.43%)
	15–17	10,714 (62.17%)
	18–19	1965 (11.40%)
Community poverty	Low	8403 (48.73%)
	High	8842 (51.27%)
Community-women educational level	Low	8870 (51.43%)
	High	8375 (48.57%)
Community contraceptive utilization	Low	7862 (45.59%)
	High	9383 (54.41%)
Country	Burundi	567 (3.28%)
	Ethiopia	832 (4.82%)
	Kenya	2169 (12.58%)
	Comoros	270 (1.56%)
	Madagascar	2117 (12.28%)
	Malawi	2734 (15.85%)
	Mozambique	2029 (11.77%)
	Rwanda	557 (3.23%)
	Tanzania	1515 (8.78%)
	Uganda	1947 (11.29%)
	Zambia	1781 (10.33%)
	Zimbabwe	728 (4.22%)

### Prevalence of teenage pregnancy in East Africa

**Fig. 1:**
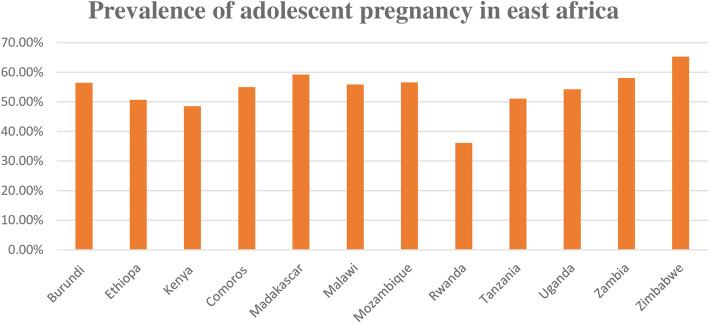
Prevalence of teenage pregnancy among adolescent girls in eastern Africa countries

The overall prevalence of teenage pregnancy in East Africa was 54.6% (95%CI; 53.85, 55.34%), ranged from 36.15% in Rwanda to 65.29% in Zimbabwe (Fig. 1).

### Factors associated with adolescent pregnancy in East Africa

#### Random effect model and model fitness

The random effect model has been assessed using ICC, MOR, and PCV. The ICC value in the null model was 0.10, which indicates that about 10% of the total variation in adolescent pregnancy was attributable due to the difference between clusters with the remaining 90% of the total variability in adolescent pregnancy was attributable due to the between-individual variability. Besides, the MOR value was 4.7 and this indicates that adolescent pregnancy was significantly different between clusters. Furthermore, PCV was highest in the final model (model III), which indicated that about 86% of the variation in adolescent pregnancy was explained by both individual and community-level factors. Regarding model fitness, the final model (Model III) was the best-fitted model for the data since it had the lowest deviance (Table 4).

**Table 4:** Random effect model and model fitness for the assessment of pregnancy among young women in eastern Africa

Parameter	Empty model	Final model
ICC	0.100677	.005415
PCV	Ref	0.865
MOR	4.7	3.4
model comparison		
Log likelihood	−11,988.704	− 9291.7749
Deviance	23,977.408	18,583.5498

#### Fixed effect analysis results

Variables including sex of household head, country and community level of education were excluded from multivariable analysis since their *p*-value at bivariable analysis was greater than 0.2. In the multivariable multilevel binary logistic regression analysis; age of respondent, marital status, relation to the household head, age at first sex, wealth status, contraceptive use, educational level, exposure to media, working status, and community level of contraceptive use were found statistically significant factors associated with adolescent pregnancy.

The odds of adolescent pregnancy among adolescent girls aged 18–19 years were 3.06 (AOR = 3.06, 95% CI: 2.83, 3.31) times higher than adolescent girls aged 15–17 years. Unmarried adolescent girls had 75% (AOR = 0.25; 95% CI: 0.23, 0.28) lower odds of being pregnant at the age of 15–19 years as compared with married adolescent girls. Adolescent girls who used contraceptives had 1.41 [AOR = 1.41; 95% CI: 1.28, 1.55] times higher odds of becoming pregnant during the adolescent period as compared to their counterparts. Besides, adolescents in the rich household had 36% (AOR = 0.64; 95% CI: 0.58, 0.71) lower odds of adolescent pregnancy as compared to adolescents from a poor household. The odds of adolescent pregnancy among adolescent girls who started sexual intercourse at the age of 15–17 years, and 18–19 years were 0.69 (AOR = 0.69; 95% CI: 0.63, 3.75), and 0.31 (AOR = 0.31; 95% CI: 0.27, 0.35) times lower as compared to those who started intercourse at the age of 5–14 years, respectively. The odds of being pregnant during the adolescent period was 1.11 times (AOR = 1.11; 95% CI: 1.03, 1.19) higher among employed girls compared to their counterparts. The odds of teenage pregnancy among adolescent girls who were spouse/head with in the family were also higher compared to being a daughter [AOR = 1.62; 95% CI: 1.45, 1.82]. Looking at media exposure, adolescent girls who had exposure to media had 0.85 times (AOR = 0.85; 95%CI: 0.78, 0.92) lower odds of being pregnant during the adolescent period compared to their counterparts. Adolescent girls with secondary and higher education had lower odds of being pregnant during adolescence compared with uneducated adolescent girls (AOR = 0.78; 95%CI: 0.68, 0.91). Regarding community-level contraceptive utilization, adolescent girls from the community with a higher level of contraceptive use had 1.10 times (AOR = 1.10; 95%CI: 1.02, 1.19) higher odds of being pregnant compared with their counterparts (Table 5).

**Table 5:** The bi-variable and multivariable multilevel binary logistic regression analysis of factors associated with adolescent pregnancy in East Africa in the final model

Variables		Teenage pregnancy		COR(95%CI)	AOR(95%CI)
		Yes	No		
**Respondent age**	**15–17**	2808	4169	1	1
	**18–19**	6607	3660	2.88 (2.70, 3.07)	3.06 (2.83, 3.31)*
**Highest educational level**	**No education**	999	429	1	1
	**Primary education**	5997	3941	0.68 (0.62, 0.76)	1.20 (1.00, 1.37)
	**Secondary and above**	2418	3458	0.31 (0.28, 0.35)	0.78 (0.68, 0.91)*
**Wealth status**	**Poor**	4511	2487	1	1
	**Middle**	1996	1443	0.77 (0.71, 0.84))	1.01 (0.91, 1.11)
	**Rich**	2909	3898	0.41 (0.38, 0.43)	0.64 (0.58, 0.71)*
**Respondent current working**	**working**	5090	3513	1.47 (1.39, 1.56)	1.11 (1.03, 1.19)*
	**Not working**	4327	4315	1	1
**Marital statues**	**Married**	6216	1796	1	1
	**Unmarried**	3200	6033	0.51 (0.14, 0.16)	0.25 (0.23, 0.28)*
**contraceptive use**	**Yes**	2550	1431	1.65 (1.53, 1.78)	1.41 (1.28, 1.55)*
	**No**	6867	6398	1	1
**Media exposure**	**Yes**	6151	5994	0.57 (0.54, 0.61)	0.85 (0.78, 0.92)*
	**No**	1835	3265	1	1
**Residence**	**Urban**	1996	2525	1	1
	**Rural**	5304	7420	1.79 (1.68, 1.92)	1.02 (0.93, 1.12)
**Relation to house hold head**	**Head/spouse**	4495	1131	6.04 (5.57, 6.57)	1.62 (1.45, 1.82)*
	**Relative or other**	2418	3004	1.15 (1.02, 1.24)	0.89 (0.82, 1.01)
	**Daughter**	2502	3605	1	1
**Age at first sex**	**5–14**	2620	1935	1	1
	**15–17**	5891	4823	0.88 (0.82, 0.95)	0.69 (0.63, 0.75)*
	**18–19**	904	1060	0.65 (0.58, 0.72)	0.31 (0.27, 0.35)*
**Community poverty level**	**Low**	3479	4018	1	1
	**High**	5937	3811	1.16 (1.09, 1.24)	1.00 (0.92, 1.07)
**Community contraceptive**	**Low**	3839	4023	1	1
	**High**	5393	3990	1.22 (1.14, 1.30)	1.10 (1.02, 1.19)*

^*^p ≤ 0.05

## Discussion

This study aimed to assess the pooled prevalence and associated factors of adolescent pregnancy in east Africa using the pooled DHS data. The pooled prevalence of teenage pregnancy in this study was 54.6% (95%CI: 53.85, 55.34%), ranging from 36.15% in Rwanda to 65.29% in Zimbabwe. It is higher than the report of previous studies [2, 4, 9]. This might be due to the difference in the study population since we have incorporated any form of pregnancy such as terminated pregnancy as teenage pregnancy, unlike other studies. The other possible explanation might be because of the large sample size and we included participants from different countries with a wide variety of socioeconomic status and cultural norms [19].

In this study; respondent age, contraceptive utilization, marital status, media exposure, respondent working status, household wealth status, community-level contraceptive utilization, age at first sex, educational level and relation to the household head were significantly associated with adolescent pregnancy. The odds of adolescent pregnancy were higher among older teenagers and this is supported by a study done in Africa [11]. This might be due to the fact that as age increases, teenagers will have more exposure to sex and their chance of getting married will also increase to procreate children [15]. Besides, older teenagers had the chance to separate from their parents and started to live independently which may lead them to have risky sexual behavior.

Surprisingly, in our study, adolescent girls who used contraceptives were at higher risk of teenage pregnancy which is in contrast to different studies [3, 19]. This may be correlated with even though there is increased use of contraception in developing countries, still a contraceptive failure, due to inadequate contraceptive counseling, awareness, and utilization skills, is common and this results in unplanned and unwanted pregnancy [17]. The higher rate of teenage pregnancy among contraceptive users also indicates that contraceptive needs may still be unmet, including intermittent use of contraceptives and supply interruption [18].

Adolescent girls from rich households had a lower risk of teenage pregnancy compared with adolescent girls from poor economic classes. This is widely accepted and in agreement with a study in Africa [19]. This may be because adolescent girls from poor households may be exposed to early marriage and sexual initiation and can’t afford the cost of reproductive health services and contraceptives [19]. Also, adolescents from families of low socioeconomic status are at greater risk of early and unintended pregnancies largely due to poverty and lower expectations of future economic success [4]. This may also be justified as young people from higher poverty levels may be involved in transactional sex as an economic survival strategy and this leads to pregnancy at a younger age [9]. In this study, early sexual initiation is a risk factor for teenage pregnancy, which is in agreement with another study done in Ethiopia [19]. This may be because women with early sexual initiation had less information, knowledge, attitude, and practice about safe sex and modern contraceptive utilization [18, 19].

The study at hand also revealed that adolescents of being spouse/head are at increased risk of teenage pregnancy, which is in line with studies in eastern Africa [4, 20]. This might be due to adolescent girls who didn’t live with both of their biological parents’ lack their parental support and guidance which exposes them to early sexual initiation, pregnancy and early motherhood [4].

Married adolescent girls had higher odds of being pregnant, which is in agreement with a study in Ethiopia [11]. Similarly, an employed adolescent girls had higher odds of teenage pregnancy compared with unemployed girls which is supported by a study done in sub-Saharan Africa [9]. This may be because adolescent girls are exposed to different risky sexual behaviors at their workplace and this sexual assault leads to pregnancy [21]. Interestingly, adolescent girls who had exposure to media had lower odds of being pregnant. This may be justified by exposure to various mass media can encourage adolescents to utilize maternal health services such as youth reproductive services and family planning services [22]. Mass media exposure also offers improved awareness and understanding, as well as improvements in attitudes, social expectations and behaviors that can contribute to beneficial effects for public health [16]. Adolescent girls who had secondary and higher education had a lower chance of being pregnant early, which is supported by a study conducted in sub-Saharan Africa [9]. This may be explained because education increases autonomy and decision-making power and increases economic independence, leading to the postponement of marriage, and reduction of fertility [23].

### Strength and limitation of the study

The study has many strengths, first, the study was based on weighted nationally representative data from 12 eastern African countries with large sample size. Second, the multilevel analysis was used to accommodate the hierarchical nature of the DHS data to get reliable standard error and estimate. Moreover, since it is based on the national survey data the study has the potential to give insight for policy-makers and program planners to design appropriate intervention strategies both at national and regional levels. However, this study had limitations in that the DHS survey was based on respondents’ self-report, this might have the possibility of recall bias. Besides, since this study was based on survey data, we are unable to show the temporal relationship between adolescent pregnancy and independent variables. Also, the independent variables for adolescent girls who gave childbirth (pregnant before) were measured at the time of the survey date.

## Conclusion

In this study, the pooled prevalence of teenage pregnancy was higher, indicating that teenage pregnancy is still the major public health problem in east Africa. Respondent’s age, contraceptive utilization, marital status, respondent working status, household wealth status, community poverty level, initiation of sex at an earlier age, residence, and relation to the household head had a significant association with teenage pregnancy. So, emphasis should be given to the reduction and prevention of pregnancy in adolescent girls to prevent adverse maternal, neonatal, educational and economic outcomes. Besides, African countries should have an integrated approach for the improvement of sexual health promotion and early pregnancy prevention among adolescent girls and women with poor socioeconomic status.

## Data Availability

All result-based data are within the manuscript and the data set is available online and anyone can access it from www.measuredhs.com.

## Abbreviations

CI: Confidence Interval
CSA: Central Statistical Agency
DHS: Demographic Health Survey
EA: Enumeration Area
ICC: Intraclass correlation
LLR: Likelihood Ratio
WHO: World Health Organization
PIH: Pregnancy-induced hypertension
PET: Preeclampsia toxemia
MOR: Median odds ratio
PCV: Proportional Change in Variance
